# Supplementary material for: Mechanisms governing the pioneering and redistribution capabilities of the non-classical pioneer PU.1
Source: Nat Commun. 2020 Jan 21;11:402. doi: 10.1038/s41467-019-13960-2 (PMC6972792; doi:10.1038/s41467-019-13960-2)
Supplement: Supplementary file 7 — Source data [file 41467_2019_13960_MOESM7_ESM.zip › Source_Data/Figure6/Figure6B_MotifScanOutput/ETS-specific/homerResults/motif11.similar.html]

motif11

## Information for motif11

G
T
C
A
T
G
A
C
G
A
C
T
G
T
C
A
G
T
A
C
T
C
G
A
G
C
T
A
G
A
C
T
A
G
C
T
G
A
T
C
G
A
T
C
G
T
A
C
  
Reverse Opposite:  

C
A
T
G
C
A
T
G
C
T
A
G
C
T
G
A
C
T
G
A
C
G
A
T
A
G
C
T
C
A
T
G
C
A
G
T
C
T
G
A
A
C
T
G
C
A
G
T
  

|  |  |
| --- | --- |
| p-value: | 1e-103 |
| log p-value: | -2.393e+02 |
| Information Content per bp: | 1.727 |
| Number of Target Sequences with motif | 363.0 |
| Percentage of Target Sequences with motif | 4.74% |
| Number of Background Sequences with motif | 354.9 |
| Percentage of Background Sequences with motif | 0.88% |
| Average Position of motif in Targets | 101.5 +/- 48.7bp |
| Average Position of motif in Background | 84.5 +/- 67.9bp |
| Strand Bias (log2 ratio + to - strand density) | 0.2 |
| Multiplicity (# of sites on avg that occur together) | 1.31 |
| Motif File: | file (matrix) reverse opposite |

### Similar de novo motifs found

|  |  |  |  |  |  |  |  |
| --- | --- | --- | --- | --- | --- | --- | --- |
| Rank | Match Score | Redundant Motif | P-value | log P-value | % of Targets | % of Background | Motif file |
| 1 | 0.971 | G T C A G T A C G A C T G T C A T G A C C T G A G C T A A G C T A G C T A T G C A G T C G A T C C T G A | 1e-103 | -239.256854 | 4.95% | 0.97% | motif file (matrix) |
| 2 | 0.943 | G T C A A G T C G A C T C G T A G T A C T C G A G C T A G A C T A G C T A G T C A G T C G A T C C T G A A C T G | 1e-101 | -234.850133 | 4.44% | 0.77% | motif file (matrix) |
| 3 | 0.813 | C G T A A G T C A G C T C G T A A G T C C T G A C G T A G C A T A C G T | 1e-93 | -214.646914 | 7.80% | 2.59% | motif file (matrix) |
| 4 | 0.936 | G T C A A G T C A G C T C G T A A G T C T C G A C G T A A C G T G A C T A G T C A G T C | 1e-92 | -213.611826 | 4.19% | 0.77% | motif file (matrix) |
| 5 | 0.883 | C G T A A G T C A C G T C G T A A T G C C T G A C G T A A G C T A G C T T A G C | 1e-76 | -176.138402 | 3.80% | 0.78% | motif file (matrix) |
